# Integrated Next Step Counseling (iNSC) for Sexual Health and PrEP Use Among Young Men Who Have Sex with Men: Implementation and Observations from ATN110/113

**DOI:** 10.1007/s10461-018-2291-2

**Published:** 2018-10-09

**Authors:** K. Rivet Amico, Jessica Miller, Christopher Balthazar, Pedro Alonso Serrano, Jennifer Brothers, Sarah Zollweg, Sybil Hosek

**Affiliations:** 1 Department of Health Behavior Health Education, School of Public Health, University of Michigan, Ann Arbor, MI USA; 2 Department of Psychiatry, Chicago, IL USA; 3 MHealthy Program, University of Michigan, Ann Arbor, MI USA

**Keywords:** PrEP, Adherence counseling, MSM, Youth, Adolescents

## Abstract

Pre-exposure prophylaxis (PrEP) for the prevention of HIV infection among young men who have sex with men is a critical part of the HIV prevention landscape in the US. Given the unique challenges and resources of young MSM negotiating safer sex practices, including PrEP, counseling and supportive discussions to optimize both PrEP use and sexual health protection more generally may facilitate reaching HIV prevention goals. Within the context of a large, open-label PrEP study (ATN110/113), support for sexual health promotion and PrEP use was provided through use of integrated Next Step Counseling (iNSC) as part of study visits. We detail iNSC and, using session documentation collected throughout this study, we characterize iNSC implementation and the content generated from these discussions. We detail features of iNSC, training of counselors and the implementation of iNSC in a multi-site PrEP study with young MSM in the US. Case report forms completed by iNSC counselors at study visits at weeks 4, 8, 12, 24, 36, and 48 were evaluated. Implementation of each intervention step for each discussion is summarized at and across timepoints, as well as features of specific steps (e.g., kinds of facilitators and barriers). Implementation differences by group (e.g., race/ethnicity, age) were examined. iNSC case report forms from 1000 sessions involving 178 unique participants ages 15–22 from sessions conducted between 2013 and 2015 were reviewed. High fidelity to iNSC steps in terms of inclusion in sessions was reported; 98–100% of sessions included critical steps for sexual health protection discussions and 96–98% for PrEP use discussions. The vast majority of sessions appeared to flow in line with iNSC’s emphasis on exploration and open discussion prior to considering specific needs and related strategies. Nearly three-quarters of sessions noted ‘commitment to staying negative’ as a motivator towards sexual health protection (more commonly reported by those identifying as White), while ‘assuming partner is negative’ was the most common challenge (less common for the older cohort), and ‘having access’ to a sexual health protection tool or strategy (besides PrEP) was the most common “need” (more common for those identifying as White or Latino). Carrying dose(s) to have them on-hand when needed was the most common PrEP adherence facilitator, drug and alcohol use was the most common challenge noted, and access to a dose when needed was the most common “need” (more common for participants self-identified as White). iNSC was implemented consistently throughout ATN110/113, and patient-centered discussions about sexual health protection and PrEP-use appeared feasible to incorporate into clinical care visits.

## Introduction

Pre-exposure prophylaxis (PrEP) is a well-supported HIV prevention strategy [1–3], approved by the United States Food and Drug Administration (FDA) since 2012 and increasingly prescribed in the US. Although rates of adherence to PrEP are estimated to be higher in demonstration projects [4] than in early placebo-controlled randomized clinical trials [5, 6], sub-optimal PrEP adherence continues to raise concerns about the potential for negative outcomes- including failure to protect from HIV and development of resistance to medications contained in PrEP, although this latter issue has been uncommon [7, 8]. The Centers for Disease Control and Prevention (CDC) [9, 10] guidelines for PrEP implementation emphasize both monitoring and support for adherence, although the evidence base for effective approaches to optimizing PrEP adherence is nascent. Various demonstration and open-label PrEP projects have adopted PrEP adherence support strategies ranging from text-message reminders to multi-session Cognitive Behavioral Therapy-based interventions, which also vary in terms of time and intensity for PrEP users and care teams alike.

In a recent open-label study within the Adolescent Trials Network (ATN) of young men who have sex with men in the United States (ATN110/113) [11, 12], we adopted a basic PrEP use support package originally developed in the iPrEX open label extension (iPrEX OLE) [13] that includes discussions about sexual health protection generally and specific to PrEP use. Integrated Next Step Counseling (iNSC) was created as a conversational, participant-centered ‘check-in’ on sexual health protection through non-biomedical and biomedical (PrEP) strategies, Implementing iNSC with adolescent and young adult men who have sex with men (MSM), we drew from well-established models of adherence that we situated within the larger socio-ecological context in which youth navigate their sexual health and well-being. In addition to comprehensive education prior to starting PrEP and instructions once PrEP is prescribed, iNSC is envisioned as a facilitated discussion of sexual health that can be implemented by clinicians or clinical care team members.

To contribute to the emerging literature that will guide PrEP providers, programs and stakeholders in their efforts to develop and implement responsive strategies to optimize PrEP adherence across diverse groups, we present iNSC and its implementation in ATN110/113.

### Integrated Next Step Counseling (iNSC)

iNSC is a two-phase discussion opened with an invitation to explore experiences and intentionally framed as a process rather than a series or set of messages. Although steps are articulated to guide implementers through the iNSC process, the driving goal is to engage participants in a non-judgmental discussion of his or her experiences surrounding protection of sexual health. Because iNSC is used in busy clinical settings with time constraints, it is implemented by interventionists with diverse levels of training. Our use of iNSC in ATN110/113 is best represented as a facilitated discussion which arguably differs from counseling (e.g., mental health counseling or therapy). We use the term ‘counseling’ here and throughout, however, to maintain consistency with protocols and projects using iNSC.

iNSC is a process for having a conversation. It draws from the Information, Motivation, Behavioral Skills model [14] situated within a socio-ecological context [15]. The kinds of experiences youth have navigating sexual health can involve personal, inter-personal, social, community-based, structural (clinic, insurer, work) and/or policy related (access to insurance, protections and rights) factors. Strategies and goals emerging from iNSC discussions can similarly span these levels and those facilitating iNSC discussions should be prepared with active referrals and ability to help PrEP users to link to wrap-around services.

### Features of iNSC

The iNSC discussion assumes that the client (used here to refer to generally to the person with whom the counselor is engaging) is the expert of his or her experiences, that experiences are influenced by multiple factors, that there are diverse pathways to adherence and engagement in sexual health that can be identified through exploration, and that facilitated exploration can lead to clients identifying their own needs and strategies. iNSC training emphasizes the importance of engaging in a genuine, as opposed to formulaic or predetermined, conversation framing the discussion around the specific context and needs of the client. Communication is intentionally neutral (non-judgmental), avoids telling clients what they must or should do, and draws on strengths, resources, and facilitators. Counselors do, however, influence the direction of the conversation moving through the steps detailed below with probing, questions and reflections.

### Specific “Steps”

**Fig. 1:**
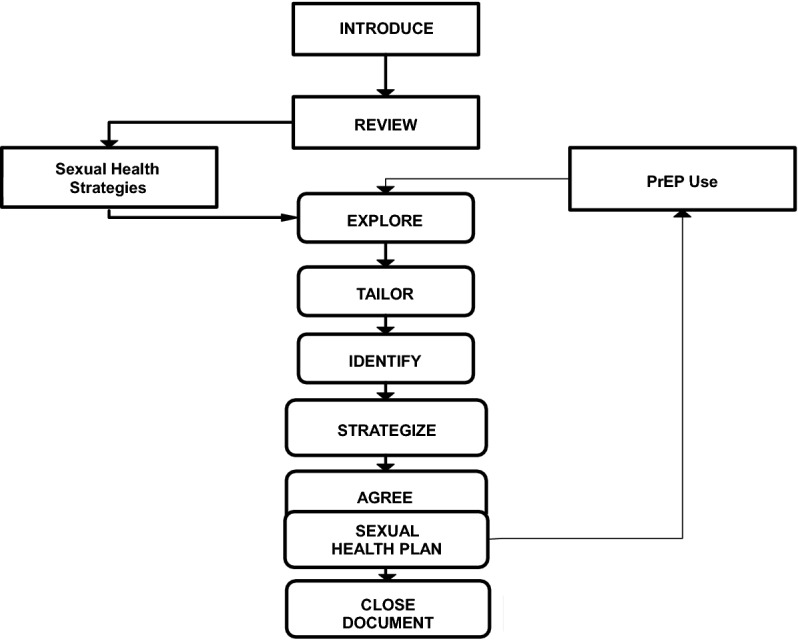
Main steps in iNSC

**Fig. 2:**
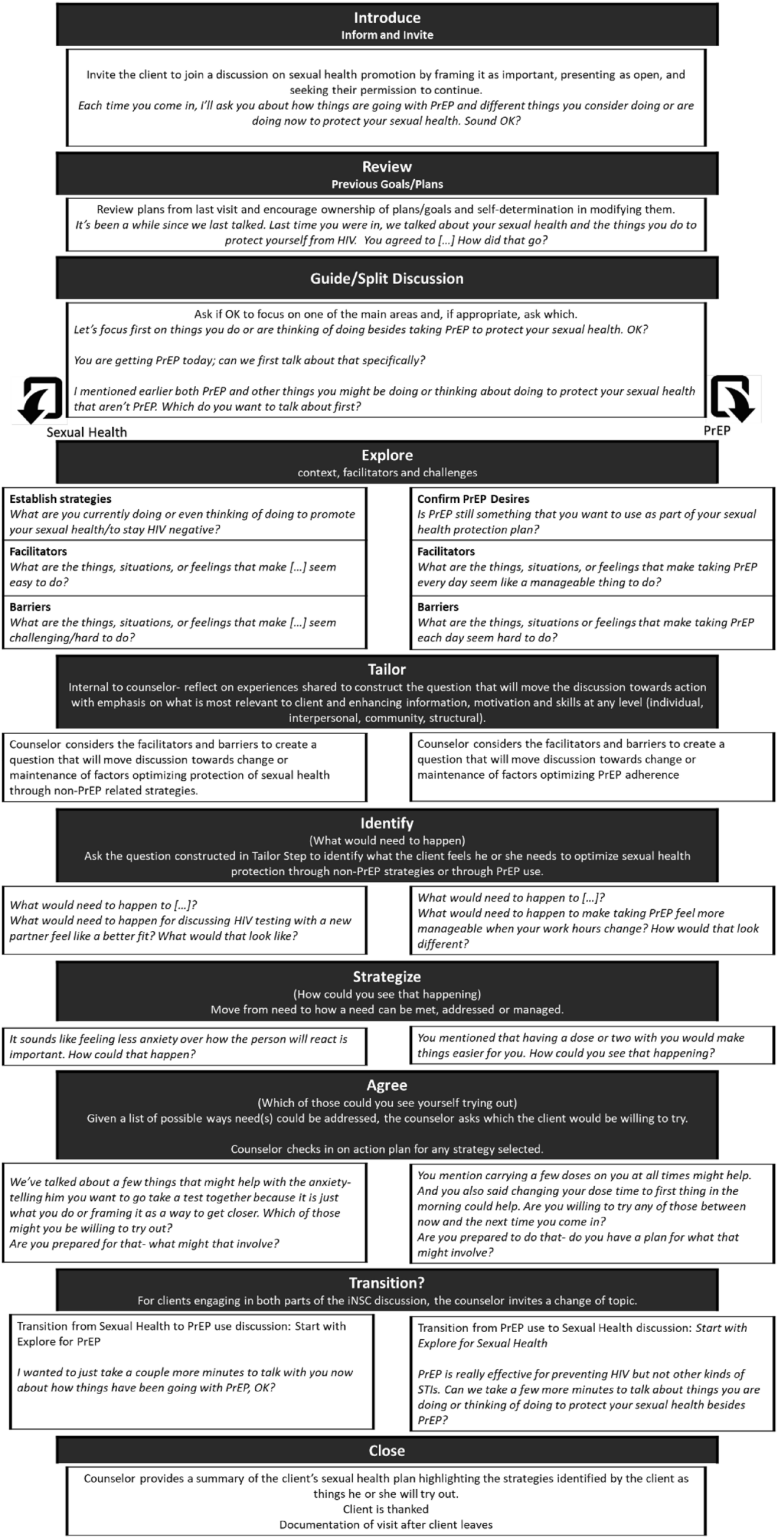
Process steps in iNSC discussion

Figures 1 and 2 depict the basic steps of iNSC. Note that we present the structure used at typical follow-up visits; education, decision-making and regimen planning components are added to first time implementation. As indicated in Fig. 1, the conversation begins with an introduction and review, and concludes with a sexual health plan. Documentation occurs after the conversation has closed and the client has left.

*Introduce* The basic feature of the introduction into the discussion is to frame what the counselor is hoping to discuss and to explicitly invite the participant to engage in the conversation. The intention is to promote shared decision making by ensuring that the participant has a voice in not only the conversation but in the decision to have the conversation. Of note, participants can decline the conversation and counselors can similarly decide *not* to implement iNSC. Typically, it is recommended that iNSC be implemented in conjunction with delivery of a negative HIV-test result, which can assist in framing the discussion.

*Review* At follow-up visits, counselors review the plan that resulted from the previous discussion and check into see if the participant worked on it and, if so, how it went. Counselors listen for whether or not the plan resonated with the participant, whether the participant engaged in it at all, what aspects seemed relevant and what aspects did not fit, and how this information might advise goal setting for the current iNSC discussion.

*Split the discussion* The focus of the conversation is then intentionally split to cover sexual health protection through non-PrEP related strategies and then, for those receiving PrEP, loops into a discussion of PrEP-use and adherence (or vice versa for those engaging in both parts of the discussion). Once the order is identified, the sequence of five steps presented below is implemented.

1. *Explore*
  This step includes asking about things, feelings, people, and situations that make the topic being discussed easy, easier, manageable or a good fit. Counselors can adapt word choices but the positioning of facilitators and/or resources within the framework suggested in Fig. 1 is important. The inquiry allows counselors to introduce the idea that many things can influence a behavior or outcome and helps participants to consider the impact of the context in which he or she tries to use PrEP or implement other prevention strategies. After exploring facilitators, counselors provide a summary and then transition to asking about the things, feelings, people and situations where the topic being discussed is difficult, challenging, uncomfortable or hard to do. A final summary is then provided of factors facilitating and factors challenging the topic being discussed.
  *For Sexual Health Protection* through non-PrEP related strategies, before exploring facilitators and challenges, the counselor inquires about which strategies the person is using or considering. The exploration then focuses on the context in which those specific or general strategies are easiest to implement and are hardest to adopt. Some clients have a long list and counselors may ask the client to focus in on the one or two strategies most meaningful to the client. Alternatively, counselors may ask about sexual health protection more globally (*What are the things that seem to help you stay motivated with using those strategies?*).
  *For PrEP*, prior to discussing facilitators and challenges, counselors confirm continued desire to remain on PrEP. If a participant no longer desires PrEP, a brief conversation about reasons and decision-making occurs, the participant is asked if he or she would discuss sexual health protection (if not already discussed), and then the counselor links the client back with the prescribing clinician. If still desired, the counselor moves into exploration of PrEP experiences.
2. *Tailor*
  After summarizing the exploration discussion, counselors prepare for the ‘Identify’ step below. To help counselors to avoid jumping immediately into solutions to the barriers just reported, they are asked to stop and think about how they will guide the discussion from here. Although this step is entirely internal to the counselor, it is an important check-point that fosters tailoring the discussion to the unique needs of the client in front of him or her. It also signals counselors to return to exploration if they find themselves unable to envision an appropriate question, probe, or reflection to use.
3. *Identify*
  Counselors ask a question at this point to direct attention to the client’s sexual health protection or PrEP use needs. Needs unique from strategies. For example, carrying doses on hand, discussing condom use with partners, or setting a timer are all strategies. Needs are *why* someone would use those strategies speaks to needs- carry doses to have them available when needed, discussing condom use to manage anxiety over rejection, or using a timer as a way to get privacy to take a dose or to help with memory. Needs reflect the underlying reason(s) for *why* someone would *do* this or that. Providing an opportunity to reflect on needs separate from barriers or strategies focuses on why someone does something (*Why would that help? How does that matter?*). There are many ways counselors can guide this segment of the discussion (see Fig. 2) while listening for what the client feels are the most causal factors at any level (self, others, clinic, policy) driving sexual health protection strategies or PrEP use.
4. *Strategize*
  After exploring needs for facilitating or maintaining sexual health protection strategies or PrEP adherence, counselors ask clients to think about how they could envision a need or needs being met or addressed. For instance, *You mentioned that managing anxiety would be key*- *how can you see that happening? What does that look like?* The client is the expert in his or her life and often can identify strategies that could work or have worked in the past in similar situations. Counselors may probe on previous experience- *Have you had a situation like this before? What helped in that situation?* Ultimately, the goal is to have multiple strategies to consider. Strategies are *not* limited to things the client can do. They may include any level in the socio-ecological framework for social determinants of health. For example, increasing stability of housing (need) to address frequent loss of PrEP pills (barrier) may require an active referral from the counselor (strategy-action plan) for linking a youth housing program (strategy). For clients reporting doing well with meeting their current needs, strategizing focuses on maintenance. Strategies may not outwardly focus on HIV risk reduction strategies (e.g., condom use, HIV-testing) or pill-taking behavior (e.g., number of doses taken or rates of adherence). Mental health, psychological well-being, substance use, housing, food access, education and employment, dealing with traumatic events, or identity development tasks may emerge as important areas that contextualize sexual health. Because the iNSC discussion approach is based on the assumption that individuals function and care for themselves in a larger context that includes all of these factors, helping clients is expected to include providing assistance in accessing resources and wrap-around services.
5. *Agree*
  With a set of strategies, the counselor asks the client which they would be willing to try before the next visit/session. For any selected strategy, the counselor checks in on an action plan- the pieces that need to fall into place for a strategy to be attempted or to work. The selected strategy or strategies become part of the sexual health protection plan. This step essentially wraps up one topic area (sexual health protection or PrEP) and the discussion can transition to the other topic area not yet covered or can move to closing the session as appropriate.

### Transition

As appropriate, after completing one topic area (e.g., sexual health protection strategies or PrEP use), counselors guide the conversation into the next topic area. For those who are not receiving PrEP but are eligible to receive it, the counselor ends the sexual health protection discussion with a confirmation of the client’s desire to continue *not* to be on PrEP and then closes or loops into a PrEP discussion if desired by the client. For those receiving PrEP, each part of the iNSC discussion is implemented.

### Close

After the appropriate topic areas have been covered, the counselor confirms the client’s sexual health plan and thanks the client for engaging in the discussion.

### Documentation

Counselors are asked to document each step of the conversation on a case report form that indicates whether or not a particular step was implemented, basic content covered for some of the steps, and strategies and goals in the client’s sexual health plan. These documents are reviewed prior to subsequent sessions to remind counselors of previous conversations. For the ATN110/113 study, these forms were maintained at site level and then collected for data entry. Counselors were asked to complete these forms after the session concluded. As counseling tools, the form represents the counselor’s recollection of the discussion content and is not cross-checked against recordings or participant recollection. Because counselors are advised that steps can be skipped or withheld from any session at their discretion and there were no immediate feedback loops in terms of supervision based on data collected on these case report forms, it is not anticipated that counselors would intentionally modify their documentation.

### Special Considerations when Implementing iNSC

Training in iNSC included segments focused on what to do when clients express clear ‘mis-information’, the importance of wording, and sessions when there are no barriers or needs reported.

*Exploration or strategizing reveals mis*-*information* In the event that a client expresses clear misinformation (e.g., *I know I need to take this only twice a week*) counselors were advised to work towards enhancing client knowledge right away. Using a strategy developed in Motivational Interviewing (MI) [16, 17] called elicit-provide-elicit, counselors first ask if the client might want to hear information about what was shared, accurate information is then provided in a neutral manner, and the counselor then asks the client what he or she makes of the new information. Given that education and re-education are essential to promoting well informed clients, most misinformation is explored immediately and then the session process resumes.

*Words matter* Counselors are encouraged to adopt word choices that demonstrate genuine curiosity, avoid blaming language, and promote multi-level exploration. For example, rather than asking “*What can you do to help you to remember your dose?*” counselors are encouraged to convey that missed doses happen in a given context and could be influenced by factors outside of the individual like, “*How could you see a situation where that dose would be taken, even when your family is visiting and you want privacy?*” Additionally, counselors are asked to vary their word choices to keep the discussions genuine and “fresh”.

*What if someone reports only facilitators?* In cases where no barriers are reported, counselors are asked to inquire about needs the person may have to “stick with” the strategies they have shared as helpful or to keep promoting the facilitators they mentioned. Counselors are asked to summarize what they heard and ask if the client feels confident in his or her ability, motivation, desire (so on) to continue with current strategies. The goal is not to force a discussion about the odd times the client’s strategies may fail, but also not to praise and race to end the discussion, given that self-report may reflect more a desire to feel praised or end the visit than reality.

### iNSC in ATN110/113

*Training* Training materials included a written iNSC implementation manual as well as videos demonstrating counseling sessions. Study site supervisors and study coordinators were trained during an in-person protocol meeting prior to the implementation of ATN 110/113. Study site staff who were responsible for counseling participants were trained by the ATN110/113 Recruitment and Counseling Coordinators, who were experienced in implementing iNSC from previous studies. Site trainings for site counselors were conducted through interactive webinars, where the counseling staff practiced live mock counseling sessions, and were reviewed live by the trainers. The webinars focused on how to use the iNSC counseling techniques to facilitate a brief and productive counseling session, but also how to respond to challenges presented by the participants. After the webinar trainings, counselors continued to practice iNSC at their sites with mock participants and submitted a video recording demonstrating their ability to conduct both a sexual health and PrEP adherence discussion using the iNSC approach. Additionally, counselors were able to receive support while strengthening their iNSC skills by site coordinators and supervisors.

*Supervision* Site counselors participated in a site by site phone call with the study Counseling Coordinator to discuss iNSC fidelity and to discuss any additional challenges related to iNSC with the study participants. Supervision started after each site enrolled its first participant and continued monthly for 5 months. Afterwards, the Recruitment and Counseling Coordinators were available for ongoing supervision of the site counselors as requested and were responsible for training and providing supervision for new staff hired during implementation of the study.

*Implementation* iNSC was generally implemented at post-HIV test delivery of a negative test. iNSC was not implemented on delivery of a positive test result. Participants in ATN110/113 starting PrEP received regimen planning discussions at first dispensation which involved further PrEP-related education, discussion of how dosing would be executed for a given participant and what to do if side-effects were experienced. At follow-up, for those on PrEP, the full iNSC process was implemented per protocol, and for those not receiving PrEP, the sexual health promotion portion was implemented.

The specific methods and outcomes of ATN110/113 have been presented [11, 18]. Here we summarize the implementation of iNSC using the case report forms completed by counselors after each iNSC session.

## Methods

*Implementation documentation* Case report forms were completed by counselors after iNSC visits and included documentation of steps included or skipped, and some details on facilitators, challenges, needs and strategies, as well as counselor rating of client’s engagement in the discussion. Each site sent their case report forms via fax and/or PDF scan uploaded to the ATN Westat FTP server and data was entered, and cross checked by the research team at Stroger Hospital. Forms were compiled and data extracted to characterize implementation in terms of steps included and details regarding some aspects of discussion content. Due to variability in data collection practices at site for this locally completed CRF, not all ATN110/113 participants had data in the compiled iNSC database. Baseline demographics for the overall sample was compared to the iNSC subset to determine if these samples may have been unique.

*Summary variables* Summary metrics were used to characterize implementation and content for facilitators, barriers and needs, and conditional frequencies were used to contrast barriers and reported needs and strategies in each topic area. Type of reported facilitators, challenges and needs over all CRFs combined were examined by groups: those who identified as Black/African American (vs. not), Latino (vs. not), White (vs. not), and age group (15–17 vs. 18–24). Differences at or above 15% between the groups compared were identified as noteworthy. Inferential statistics with probability levels were not used. Rather, the differences are noted as a characterization of potential unique aspects of iNSC conversations in various demography groups.

*Human subjects protection* ATN110/113 procedures were approved and monitored throughout the conduct of the trial through the Adolescent Trials Network. Procedures, consent forms, and study materials were approved by each participating clinical research site’s regulatory boards.

## Results

### Sample

A total of 24 counselors across 10 research sites implemented iNSC over the course of the ATN110/113 study, from January 2013 to November 2015. Information about implementation of each iNSC session was collected on a study case report form (CRFs) and forms were collected from each participating site at the close of the study.

A total of 1029 case report forms were collected. From these, a total of 1000 were identified as containing any information about iNSC implementation over entry to 48 weeks of being on study. The 178 unique participants represented in the iNSC data set were ages 15–22 (mean age 19, SD = 2.12) with the majority self-identifying as gay (60%). Slightly over half identified as Black/African American, 23% Hispanic/Latino, and 10% White. Over a quarter (27%) had their GED or High School diploma, and 40% had part or full-time employment at baseline. Many (44%) lived with family/parents, and very few (< 1%) reported living on the street. No differences were noted between the iNSC included sample and baseline demography from participants not captured in the iNSC data set.

### Implementation of iNSC

As indicated in Table 1, CRFs captured the implementation of six critical steps in iNSC (introduce, review, explore, identify needs, strategize, agree on a strategy to try) at each visit and specific to parts of the discussion focused on sexual health protection and PrEP use. For each step, the overall percent of sessions that included the step all exceeded 95% in each focus area (sexual health and PrEP use). Ratings of level of engagement in the discussion were characterized as percent marked in the highest rating (most engaged) in the Tailor step. These suggested the vast majority were highly engaged in the discussions (73% of sessions were rated as highest level of engagement in sexual health discussions and 78% in PrEP discussions over all iNSC sessions). About 82% of sessions had needs successfully identified and discussed in each focus area (sexual health and PrEP use).

**Table 1:** Implementation of iNSC over all documented sessions

	Prior to week 4	Week 4	Week 8	Week 12	Week 24	Week 36	Week 48	Total
Introduce step	221/224 (99%)	141/141 (100%)	138/138 (100%)	137/139 (99%)	133/134 (99%)	118/118 (100%)	106/106 100%)	994/1000 (96%)
Review step	NA	140/141 (99%)	138/138 (100%)	136/139 (98%)	133/134 (99%)	115/118 (98%)	106/106 (100%)	768/776 (99%)

	Prior to week 4		Week 4		Week 8		Week 12		Week 24		Week 36		Week 48		Total	
Focus	SH	PrEP	SH	PrEP	SH	PrEP	SH	PrEP	SH	PrEP	SH	PrEP	SH	PrEP	SH	PrEP
Explore Step	222/224 (99%)	130/148 (88%)	141/141 (100%)	138/138 (100%)	138/138 ((100%)	135/135 (100%)	139/139 (100%)	132/135 (98%)	134/134 (100%)	128/130 (99%)	118/118 (100%)	107/107 (100%)	106/106 (100%)	97/99 (98%)	998/1000 (100%)	867/892 (97%)
Identified at least 1 facilitator	214/222 (96%)	126/129 (98%)	214/222 (96%)	136/138 (99%)	135/138 (98%)	131/135 (98%)	131/136 (96%)	128/132 (97%)	129/133 (97%)	123/127 (97%)	112/118 (95%)	103/107 (96%)	95/103 (92%)	87/96 (91%)	949/991 (96%)	834/892 (97%)
Identified at least 1 challenge	186/219 (85%)	90/128 (70%)	106/139 (76%)	118/138 (86%)	103/136 (76%)	110/135 (82%)	103/136 (76%)	108/131 (83%)	96/132 (73%)	110/127 (86%)	77/118 (65%)	81/106 (76%)	67/105 (64%)	72/96 (75%)	738/985 (75%)	689/861 (80%)
Tailor (% High)	161/224 (72%)	122/148 (82%)	108/141 (78%)	106/138 (77%)	100/138 (73%)	100/135 (74%)	103/139 (74%)	105/135 (78%)	92/134 (69%)	96/130 (74%)	87/118 (74%)	85/107 (79%)	28/106 (77%)	78/99 (79%)	733/1000 (73%)	692/892 (78%)
Identify Step (What)	219/224 (99%)	129/148 (87%)	140/141 (99%)	137/138 (99%)	136/138 (99%)	134/135 (99%)	136/139 (98%)	132/135 (98%)	130/134 (97%)	125/130 (96%)	118/118 (100%)	106/107 (99%)	106/106 (100%)	96/99 (97%)	985/1000 (99%)	859/892 (96%)
Identified at least 1 need	192/219 (88%)	104/129 (81%)	120/140 (86%)	119/137 (87%)	112/136 (82%)	112/135 (84%)	111/136 (82%)	110/132 (83%)	103/130 (79%)	105/125 (84%)	88/118 (75%)	79/106 (75%)	70/106 (75%)	74/96 (77%)	806/985 (82%)	703/859 (82%)
Strategize Step (How)	221/224 (99%)	129/148 (87%)	139/141 (99%)	137/138 (99%)	136/138 (98%)	134/135 (98%)	136/139 (98%)	132/135 (98%)	131/134 (98%)	126/130 (97%)	117/118 (99%)	106/107 (99%)	105/106 (99%)	95/99 (96%)	985/1000 (99%)	859/892 (96%)
Agree Step (Which)	218/224 (97%)	129/148 (87%)	139/141 (99%)	137/138 (99%)	135/138 (98%)	132/135 (98%)	135/139 (97%)	132/135 (98%)	131/134 (98%)	126/130 (97%)	117/118 (99%)	106/107 (99%)	104/106 (98%)	95/99 (96%)	979/1000 (98%)	858/892 (96%)

*SH* sexual health protection through non-PrEP related strategies, *PrEP* PrEP use/adherence support

### iNSC Content

*Sexual health protection focus* Counselors marked and characterized facilitators, challenges, and needs from each session. Not being able to identify a facilitator, challenge or need was a valid option in the form and counselors were encouraged to mark this if discussions were introduced and exploration did not identify anything particular to “work on” in the session. At least one sexual health protection facilitator was marked as identified in 96% of sessions, one challenge in 75% of discussions and at least one specific need to discuss in 82%. Content or type of facilitator, barrier, and need(s) are listed in Table 2. Across the 1000 documented CRFs, the most commonly noted facilitators to sexual health included personal commitment (motivation) to stay negative (74%) and confidence in negotiating strategies with sex partner (34%). The most common sexual health challenges included thinking partners are HIV-negative without really knowing their status (28%) and being caught up in the moment (24%). Most commonly noted needs included needing to have access to a strategy when needed or wanted (condoms, testing, HIV testing, and lube) (51%), being assertive and confident (16%), and having better concrete skills around negotiating strategies with partners (14%). On average, the total number of facilitators, challenges, and needs noted was 1.

**Table 2:** Facilitators, challenges and needs in sexual health iNSC discussions

	N = 1000	%
**Sexual health facilitators**		
Personal commitment (motivation) to stay negative^a^	739	73.90
Confidence in negotiating strategies with sex partner	337	33.70
Being well informed^b^	257	25.70
Partner supports strategies	221	22.10
Fits well into what I do sexually	161	16.10
Having intimacy with my partner(s)	150	15.00
Other	48	4.80
None identified	42	4.20
Not discussed	7	0.70
**Sexual health challenges**		
Thinking partners are HIV-negative without really knowing their status^c^	281	28.10
None could be identified	247	24.70
Caught up in the moment	241	24.10
Interferes with intimacy	122	12.20
Partner(s) unwilling/reluctant/against to practice strategies	101	10.10
Fearful of rejection or missed opportunity (ruining the mood)	96	9.60
Feeling down/sad (not caring about protecting self)^d^	93	9.30
Other	83	8.30
Drug or alcohol use (making decisions difficult)^e^	77	7.70
Not feeling well informed	57	5.70
Specific incentives to not use strategies (pay or trade)	16	1.60
Not thinking that getting HIV would be bad	12	1.20
Not discussed	5	0.50
**Sexual health needs**		
Have access to strategies (condoms, HIV testing, lube)^f^	514	51.40
None could be identified	179	17.90
Be assertive/confident	159	15.90
Have better concrete skills around negotiating strategies with partners	142	14.20
Social support	103	10.30
Have strategies that are sexy/fit into sexual life	97	9.70
Feel more motivated	92	9.20
Feel better informed	85	8.50
Other	66	6.60
Basic living needs met (housing, food, safety)	44	4.40
Not discussed	8	0.80

^a^Youth identifying as White had more sessions with this facilitator (90% vs. 74%)

^b^Youth identifying as Latino had fewer sessions with this facilitator (15% vs. 31%)

^c^Older youth had fewer sessions with this challenge (24% vs. 42%)

^d^Youth identifying as Latino had more sessions with this challenge (23% vs. 5%)

^e^Youth identifying as White had more sessions with this challenge (25% vs. 5%)

^f^Youth identifying as White had more sessions with this challenge (69% vs. 53%), as did those identifying as Latino (66% vs. 50%)

*PrEP use focus* Specific to the portion of the discussion focused on experiences with PrEP, at least one PrEP use related facilitator was marked as identified in 97% of sessions, one challenge in 80% of discussions, and at least one need to discuss in 82%. The main facilitators, barriers and needs identified are listed in Table 3. Across the 892 CRFs that explored PrEP, the most commonly reported facilitators were dosing aids and tools (e.g. calendar, alarms) (83%), matching with routine or events (38%) and commitment to protecting self and others (29%). PrEP challenges most commonly noted included partying/drugs/alcohol (32%), medication related concerns (e.g. too big, taste bad) (28%), and disruption in routine (27%). Most common needs included access (have medication available) (58%) and remembering to take the medication (20%). On average, the total number of facilitators, challenges, and needs noted was 1.

**Table 3:** Facilitators, challenges and needs in PrEP iNSC discussions

	N = 892	%
**PrEP facilitators**		
Mobile/carry tools (e.g. pill boxes)	739	82.85
Match with routine/event	337	37.78
Commitment/protecting self or others	257	28.81
Memory aids/tools (e.g. calendar, alarm)	221	24.78
Access	161	18.05
Social support (family, friends, partners)	150	16.82
Other	48	5.38
None could be identified	42	4.71
Not discussed	7	0.78
**PrEP challenges**		
Partying/drugs/alcohol	281	31.50
Medication (too big, taste bad)	247	27.69
Disruption in routine	241	27.02
Forgetting/no dose available	122	13.68
Side effects	101	11.32
Lack of privacy	96	10.76
Other	93	10.43
None could be identified	83	9.30
Not discussed	77	8.63
**PrEP needs**		
Access (have available)^a^	514	57.62
Remember	179	20.07
Motivation	159	17.83
Manage side effects	142	15.92
Privacy	103	11.55
Social support	97	10.87
Other	92	10.31
None could be identified	85	9.53
Not discussed	66	7.40

^a^Youth identifying as White had more sessions with this need (57% vs. 38%)

*Groups differences* Total numbers of facilitators, challenges and needs noted in sexual health discussions did not differ by group membership. iNSC sexual health discussion documentation with Black/African American (vs. not) youth did not markedly differ on content. The only differences (15% or greater difference) in endorsed types of sexual health facilitators, challenges or needs were more sessions with White identified youth having ‘personal commitment to stay HIV negative’ (90% vs. 74%) as a facilitator, drug and alcohol use (25% vs. 5%) as a challenge, and needing to have access to sexual health protection strategies (69% vs. 53%); fewer Latino identified youth reported feeling well informed (15% vs. 31%) as a facilitator and more with ‘feeling down or sad’ (23% vs. 5%) as a challenge and needing to have access to sexual health protection strategies (66% vs. 50%). More participants in the younger group had “thinking partner(s) is HIV negative” (42% vs. 24%) as a challenge.

Specific to PrEP, youth self-identifying as White had greater representation in having a need for ‘access to a PrEP dose’ (57% vs. 38%). No other differences between demography groups at or exceeding 15% were identified in types of PrEP facilitators, challenges or needs.

## Discussion

Results provide support for the feasibility of implementing iNSC in the context of this research project. According to post-session documentation completed by counselors, each step of the counseling approach was consistently implemented. Content captured in the case report forms suggested that challenges reported did not always match the “needs” being worked on (e.g., what the client felt was most important to work on with the counselor at that time). In developing and adapting iNSC to context-driven, individually-tailored, adolescent focused discussions, we hypothesized that providing a specific focus on exploring one’s context would optimize the quality and depth of discussion. While we cannot evaluate this directly, content from the collected CRFs do support a complex picture of the context in which sexual health and PrEP use was experienced. With no control condition, however, the effect of iNSC in comparison to different approaches or even in comparison to no conversation at all is unknown.

Results from ATN 110 (open-label PrEP among MSM 18–22 years of age) reported high levels adherence (≥ 4 pills per week) in the majority of participants (56%) at their 4-week study visit estimated via TFV-DP levels, which decreased over time to 34% with drug concentrations consistent with high levels of PrEP protection at their 48-week visit [18]. Findings for ATN113, involving at risk MSM 15–17 years of age, showed similar patterns of starting strong (60% with highly protective levels at week 4 dropping to 28% with high levels at week 48) [18]. There was a noticeable drop in adherence when youth were transitioned from monthly to quarterly contacts with the study team, which included implementation of iNSC. Although observational, it is possible that extending the time between contacts may have eroded adherence among young MSM. Future work to determine optimal visit schedules for young MSM receiving PrEP and the components of these visits that would facilitate high PrEP adherence and persistence is needed.

Observational data, collected through second person (counselor completed CRFs) limits conclusions that can be drawn from the current research. With the very high rates of implementation for each step in iNSC and specific themes in content recorded, iNSC did appear to have high level of feasibility from an implementation standpoint. We cannot speak to the quality of implementation, which is a critical determinant of potential impact. Future work using iNCS should include opportunities for direct observation through recorded sessions or other methods that allow for fidelity assessments, feedback, and ongoing training. Additionally, whether clients or participants found the approach acceptable was not directly assessed. Other qualitative work with participants reported positive overall sentiments about counselors and interactions with the clinical care team [12], which should be considered in overall feasibility and acceptability. Finally, our data does not speak to potential impact of sessions, generally or those that focused on specific content on drug levels or reported PrEP adherence. Future work is needed to determine if the iNSC approach is effective over alternative approaches.

Importantly, iNSC was developed largely for those who are at least provisionally aligned with trying to use PrEP. In situations where individuals are skeptical or have reservations, discussions should focus on decision making and exploring factors influencing beliefs about and decisions around PrEP start. Ultimately, any discussion-based approach requires open discourse. If individuals cannot openly share use or non-use, concerns or fears about PrEP, any discussion-based approach will suffer. Thus, efforts to align individuals are critical preliminary steps to consider prior to any discussions that focus on adherence or persistence (cf., [19]).

As PrEP is rolled out in the US, strategies to create opportunities to discuss sexual health and PrEP use, included at each clinic visit, should be considered. Even brief conversations positioned with delivery of HIV-negative test results during routine PrEP visits may be able to hone in on contextual, interpersonal, and personal factors influencing one’s experiences with sexual health and PrEP use. If implemented in a safe and supportive context, these genuine conversations can help to create the kinds of welcoming and inclusive environments that would promote ongoing engagement. Additional implementation research is needed to best tailor interventions to the specific needs of young MSM in the US and globally.
